# New Appliances and Things Medical

**Published:** 1893-11-25

**Authors:** 


### NEW APPLIANCES AND THINGS MEDICAL.

### SCOTCH OATMEAL.

(John Inglis and Sons, Leith.)

Messrs. Inglis have sent us a sample of their oatmeal, which is of most excellent quality. There is a far greater difference between the quality of different oatmeals than is generally supposed; yet the difference is very apparent where a practical test is applied. Some meals we have seen are barely crushed grains, whilst others are very little less descicated than wheaten flour. Either of these descriptions is equally objectionable to the connoisseur of oatmeal, and are wholly different to Messrs. Inglis's carefully-prepared speciality. The "Midlothian Oatmeal" leaves nothing to be desired, individual tastes being provided for by a supply of five degrees of coarseness. Messrs. Inglis are purveyors to the Prince of Wales. Their London agents are John Alexander and Co., 61, Mark Lane.

